# Hemolysis After Transcatheter Pulmonary Valve Implantation Associated With Residual Shunt Flow

**DOI:** 10.1016/j.jaccas.2026.109644

**Published:** 2026-08-04

**Authors:** Taro Hayashi, Teruhiko Imamura, Shuhei Tanaka, Hideyuki Nakaoka, Keijiro Ibuki, Hiroshi Ueno, Koichiro Kinugawa

**Affiliations:** a Second Department of Medicine, University of Toyama, Toyama, Japan; b Department of Pediatrics, University of Toyama, Toyama, Japan

**Keywords:** chronic heart failure, congenital heart defect, pulmonary valve, ventricular septal defect

## Abstract

**Background:**

Transcatheter pulmonary valve implantation (TPVI) is an established treatment for severe pulmonary regurgitation after repaired tetralogy of Fallot, whereas clinically significant hemolysis is rare.

**Case Summary:**

A 39-year-old man with repaired tetralogy of Fallot developed severe pulmonary regurgitation with progressive right ventricular enlargement. TPVI using a self-expandable Harmony valve was successfully performed. Postprocedural dark-red urine and laboratory findings confirmed hemolysis. Multimodality imaging revealed a coronary artery–right ventricular fistula and residual ventricular septal defect adjacent to the valve. High-velocity shunt flow impinging on the prosthesis was considered the mechanism. Hemolysis resolved with blood pressure control alone.

**Discussion:**

Residual shunt lesions adjacent to the right ventricular outflow tract may cause mechanical hemolysis after TPVI by generating localized high-shear stress. This case highlights the importance of comprehensive multimodality imaging before TPVI.

**Take-Home Message:**

Residual shunt flow adjacent to the right ventricular outflow tract may cause mechanical hemolysis after TPVI.

## Visual Summary

**Figure:**
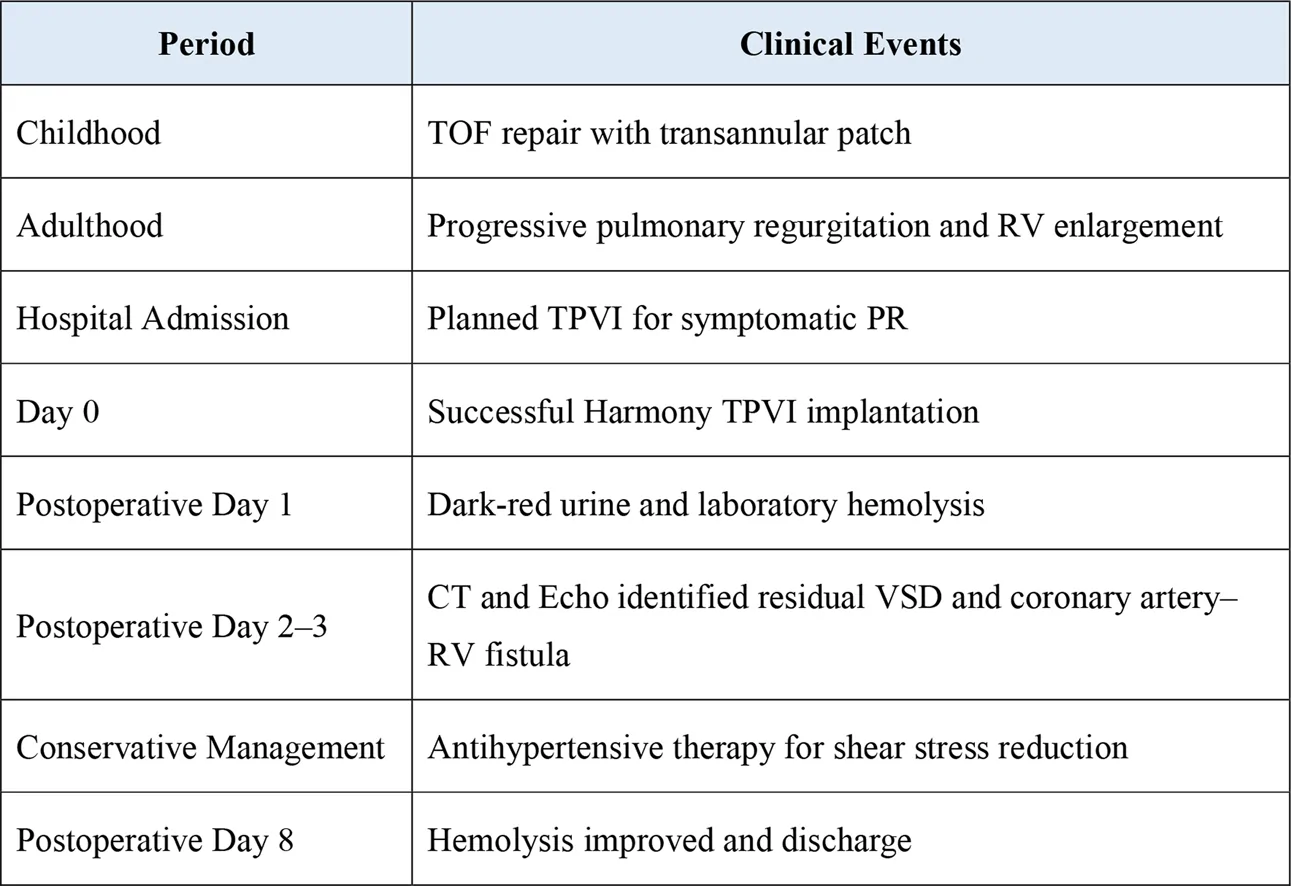

Tetralogy of Fallot (TOF) is one of the most common cyanotic congenital heart diseases, consisting of ventricular septal defect (VSD), overriding aorta, right ventricular outflow tract (RVOT) obstruction, and right ventricular hypertrophy.^1^ Surgical intracardiac repair during infancy has dramatically improved long-term survival.^2^ However, patients who undergo transannular patch repair frequently and gradually develop chronic pulmonary regurgitation later in life because pulmonary valve tissue is partially or completely resected to relieve RVOT obstruction.^3^

Chronic pulmonary regurgitation may lead to progressive right ventricular dilation, ventricular arrhythmias, exercise intolerance, heart failure, and sudden cardiac death.^4^ Accordingly, pulmonary valve replacement is recommended in symptomatic patients or those with progressive right ventricular enlargement or dysfunction.^5^

Recently, transcatheter pulmonary valve implantation (TPVI) has emerged as a less invasive alternative to surgical pulmonary valve replacement. The development of self-expandable valves has enabled successful TPVI in patients with large or patch-repaired RVOTs.^6^ Although TPVI generally demonstrates high procedural success and favorable mid-term outcomes, complications such as infective endocarditis, valve thrombosis, and stent fracture have been reported.^6^, ^7^, ^8^ In contrast, clinically significant hemolysis after TPVI has rarely been described.

We herein report a case of mechanical hemolysis after TPVI in a patient with repaired TOF, in whom a coronary artery–right ventricular fistula and residual VSD were considered potential contributors.

## Case Presentation

### Patient history

A 39-year-old man with repaired TOF was admitted for TPVI. He had undergone intracardiac repair with transannular patch repair at 3 years of age. Residual VSD and pulmonary regurgitation persisted after surgery, and he had experienced exertional dyspnea and lower extremity edema requiring diuretic therapy. One year before admission, cardiac magnetic resonance imaging demonstrated severe right ventricular enlargement with a right ventricular end-diastolic volume index of 185.8 mL/m^2^. In addition, paroxysmal atrial tachycardia had been treated with catheter ablation at our institution. Nonetheless, exertional dyspnea persisted, and TPVI was planned. Medications included apixaban 5 mg, candesartan 4 mg, furosemide 20 mg, and febuxostat 10 mg daily.

### Physical examination and laboratory findings

On admission, his height was 157.2 cm, body weight was 65.8 kg, and body mass index was 26.7 kg/m^2^. Blood pressure was 114/62 mm Hg, pulse rate was 76 beats/min, and oxygen saturation was 98% on room air. Chest radiography showed cardiomegaly with a cardiothoracic ratio of 58% (Figure 1A). Electrocardiography demonstrated sinus rhythm with complete right bundle branch block (Figure 1B). Laboratory testing revealed hemoglobin 13.8 g/dL, serum creatinine 0.70 mg/dL, serum total bilirubin 0.5 mg/dL, and plasma B-type natriuretic peptide 154 pg/mL.

**Figure 1:**
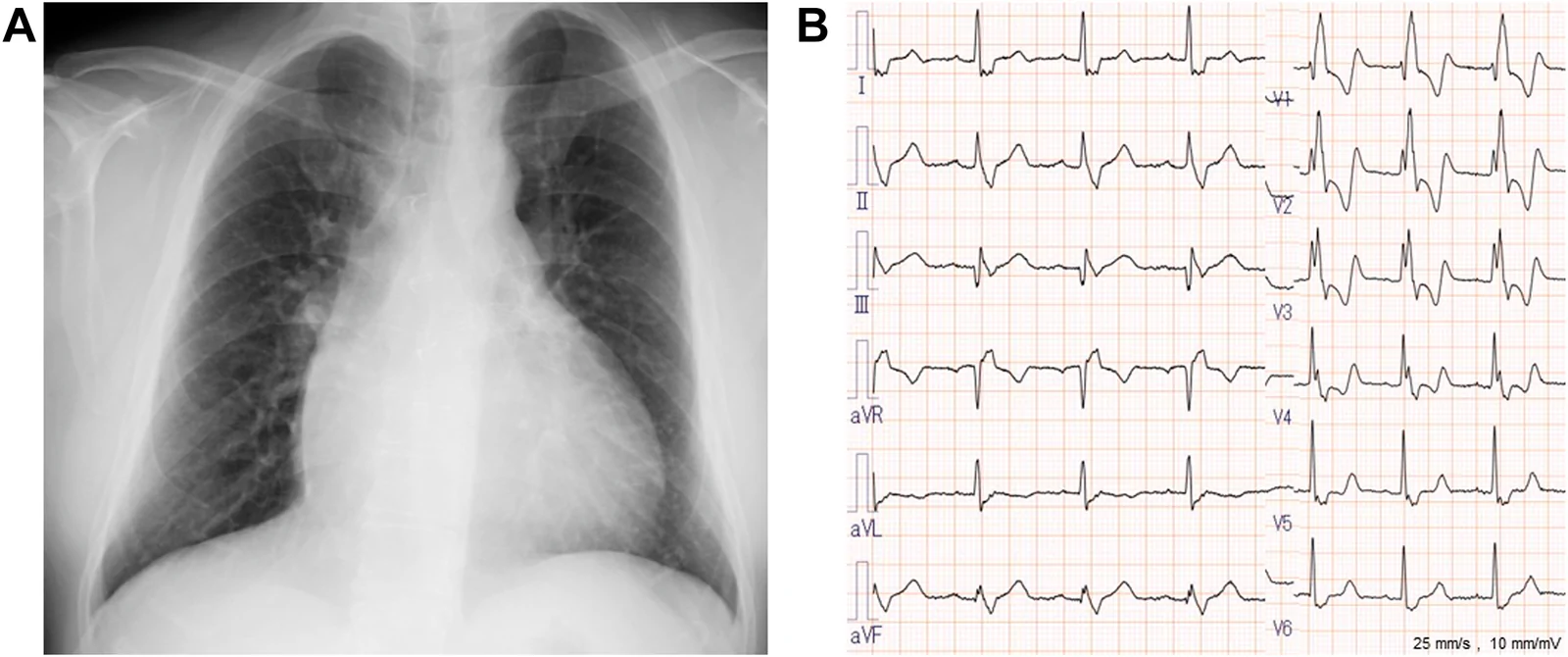
Baseline Chest Radiography and Electrocardiography (A) Chest radiography on admission demonstrating cardiomegaly with a cardiothoracic ratio of 58%. (B) 12-lead electrocardiography showing sinus rhythm with complete right bundle branch block.

### Cardiac imaging

Transthoracic echocardiography demonstrated moderate pulmonary regurgitation (Figure 2A), preserved left ventricular ejection fraction (52%), and right ventricular pressure overload with a D-shaped left ventricle (Figure 2B). Cardiac magnetic resonance imaging confirmed severe right ventricular enlargement and residual VSD (Figure 2C). The residual VSD was considered small and was not regarded as the primary cause of the patient's progressive right ventricular enlargement, which was mainly attributed to severe pulmonary regurgitation. Right heart catheterization showed mean right atrial pressure of 12 mm Hg, right ventricular end-diastolic pressure of 15 mm Hg, and mean pulmonary artery pressure of 18 mm Hg.

**Figure 2:**
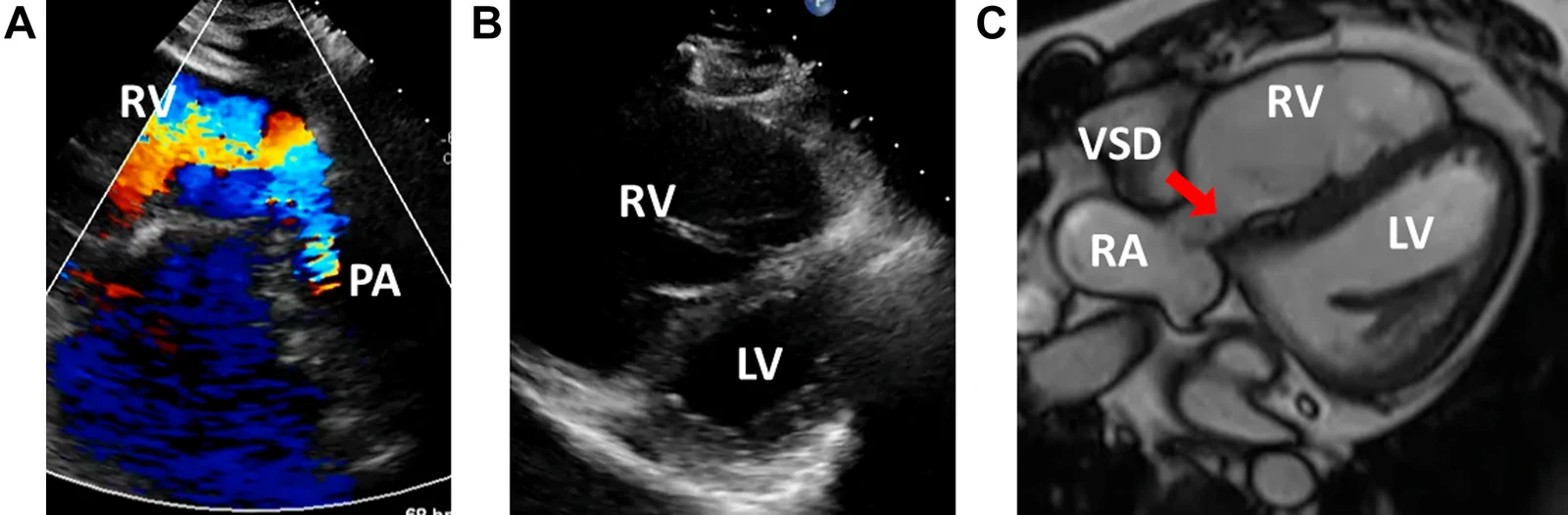
Preprocedural Cardiac Imaging Findings (A) Transthoracic echocardiography demonstrating severe pulmonary regurgitation. (B) Parasternal short-axis echocardiographic view showing a D-shaped left ventricle, suggesting right ventricular pressure overload. (C) Cardiac magnetic resonance imaging demonstrating severe right ventricular enlargement and residual VSD. LV = left ventricle; PA = pulmonary artery; RA = right atrium; RV = right ventricle; VSD = ventricular septal defect.

### Transcatheter pulmonary valve implantation

TPVI using a self-expandable Harmony transcatheter pulmonary valve (Medtronic Minneapolis) was successfully performed via the femoral vein approach. Severe pulmonary regurgitation markedly improved after valve implantation without immediate procedural complications (Figures 3A to 3D).

**Figure 3:**
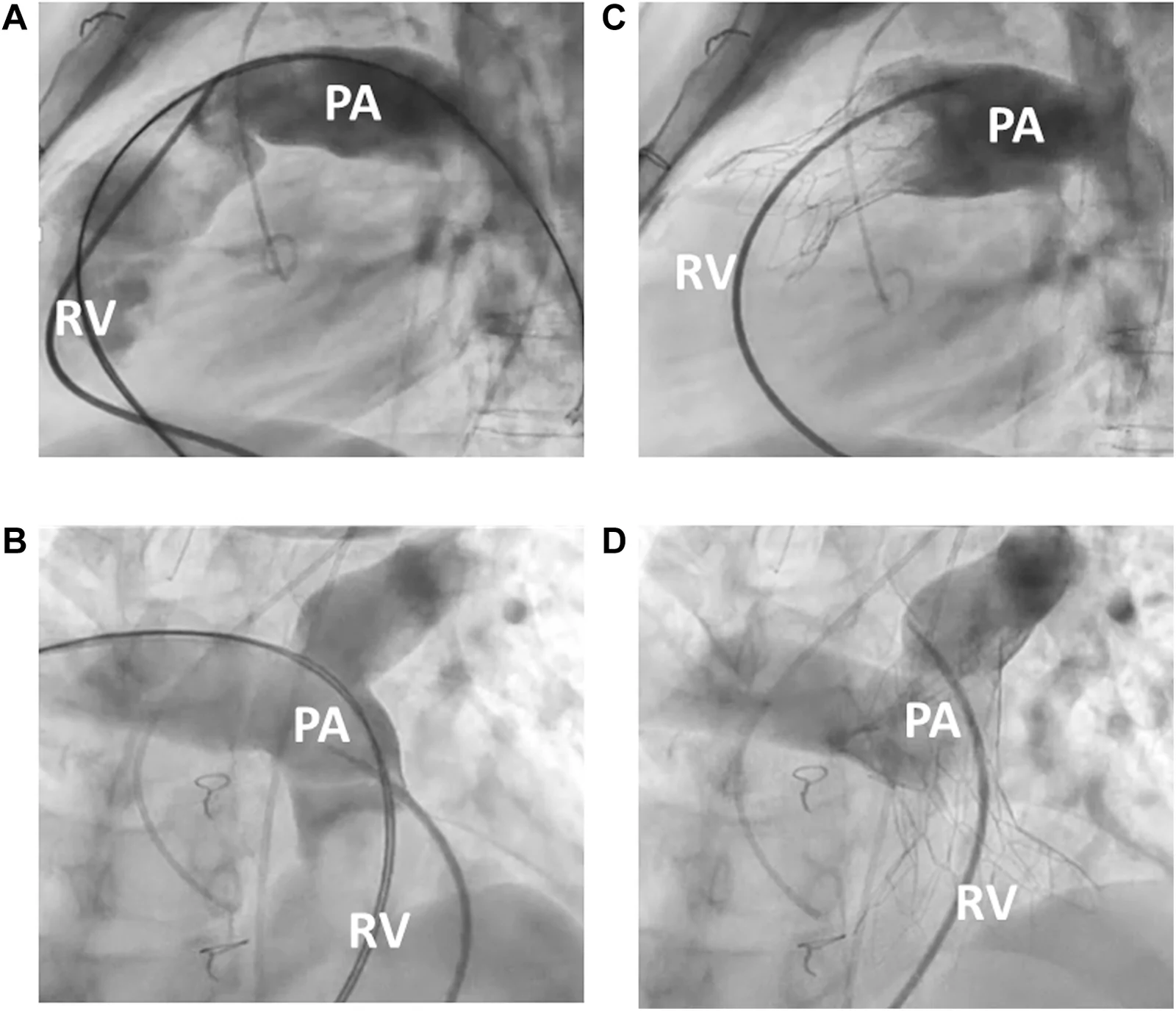
Transcatheter Pulmonary Valve Implantation Procedure (A and B) Angiographic image before transcatheter pulmonary valve implantation demonstrating severe pulmonary regurgitation. (C and D) Angiographic image after implantation of a self-expandable Harmony valve showing marked reduction of pulmonary regurgitation. PA = pulmonary artery; RV = right ventricle.

### Development of hemolysis

During the night after the procedure, the patient developed dark-red urine (Figure 4). Laboratory testing demonstrated marked elevation of lactate dehydrogenase and bilirubin levels with reduced haptoglobin (6 mg/dL), suggesting intravascular hemolysis. Hemoglobin levels progressively decreased after TPVI.

**Figure 4:**
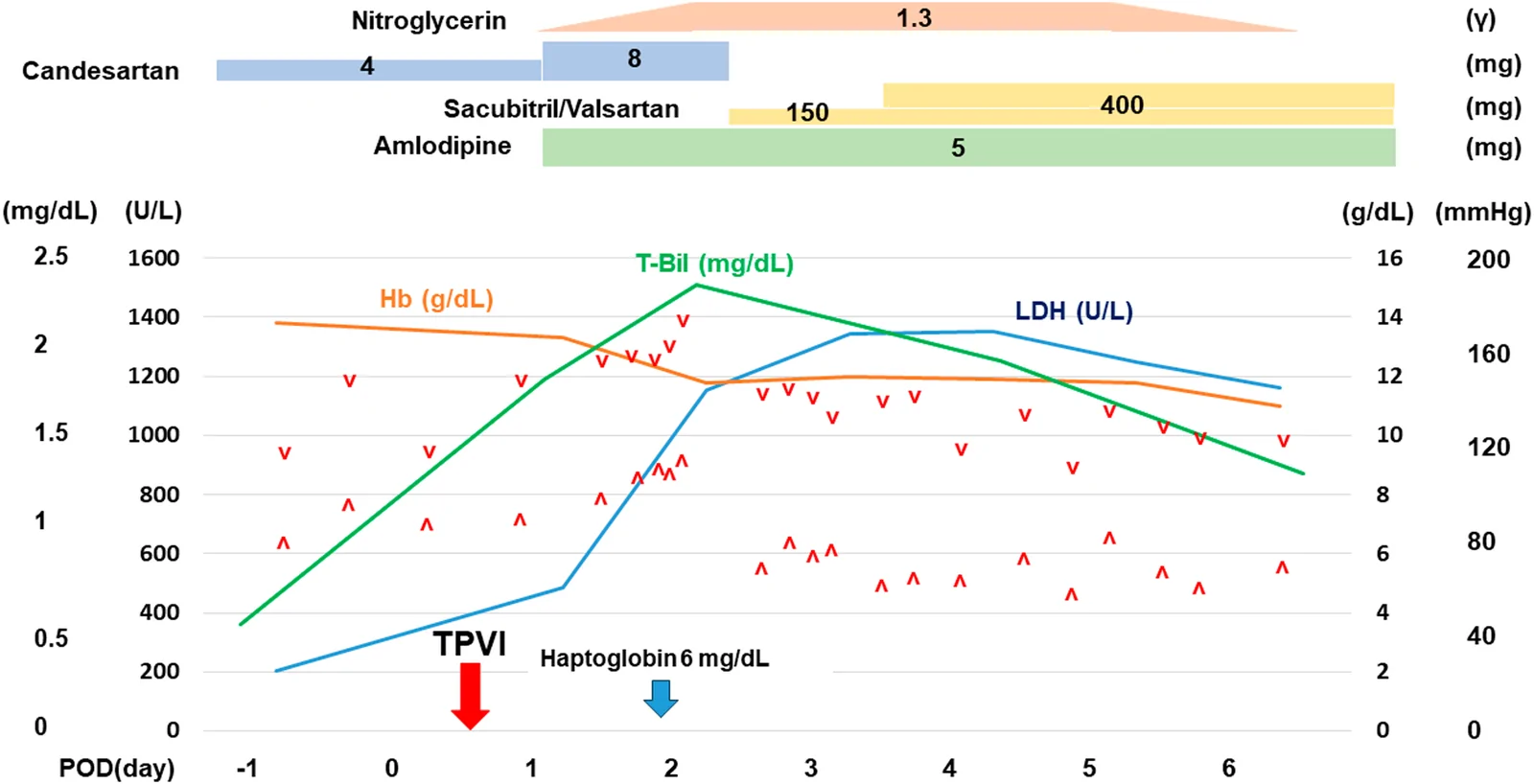
Clinical Course After Transcatheter Pulmonary Valve Implantation Following TPVI, laboratory findings demonstrated progressive hemolysis with elevation of LDH and T-Bil levels, accompanied by a decrease in Hb. Serum haptoglobin level decreased to 6 mg/dL. Antihypertensive therapy was initiated to reduce mechanical shear stress. Hemolytic findings gradually improved during conservative management. Hb = hemoglobin; POD = postoperative day; T-Bil = total bilirubin; TPVI = transcatheter pulmonary valve implantation.

Mechanical hemolysis related to paravalvular leakage was initially suspected, and antihypertensive therapy with candesartan, sacubitril/valsartan, amlodipine, and intravenous nitroglycerin was initiated to reduce shear stress.

### Additional imaging and clinical course

Subsequent cardiac computed tomography demonstrated a coronary artery–right ventricular fistula (Figures 5A and 5B) and residual VSD near the implanted valve (Figure 5C). A branch arising from the proximal left anterior descending artery (segment #7), distal to the origin of the left circumflex artery, coursed toward the right ventricle and drained into the RVOT, forming a coronary artery–right ventricular fistula. The fistula was located directly adjacent to the upper margin of the first diamond cell from the inflow end of the implanted Harmony valve. Transthoracic echocardiography demonstrated 2 accelerated flows around the RVOT adjacent to the implanted valve (via coronary fistula and VSD) (Figure 6A and 6B). High-velocity shunt flow directed toward the prosthetic valve structure was considered the likely mechanism of hemolysis.

**Figure 5:**
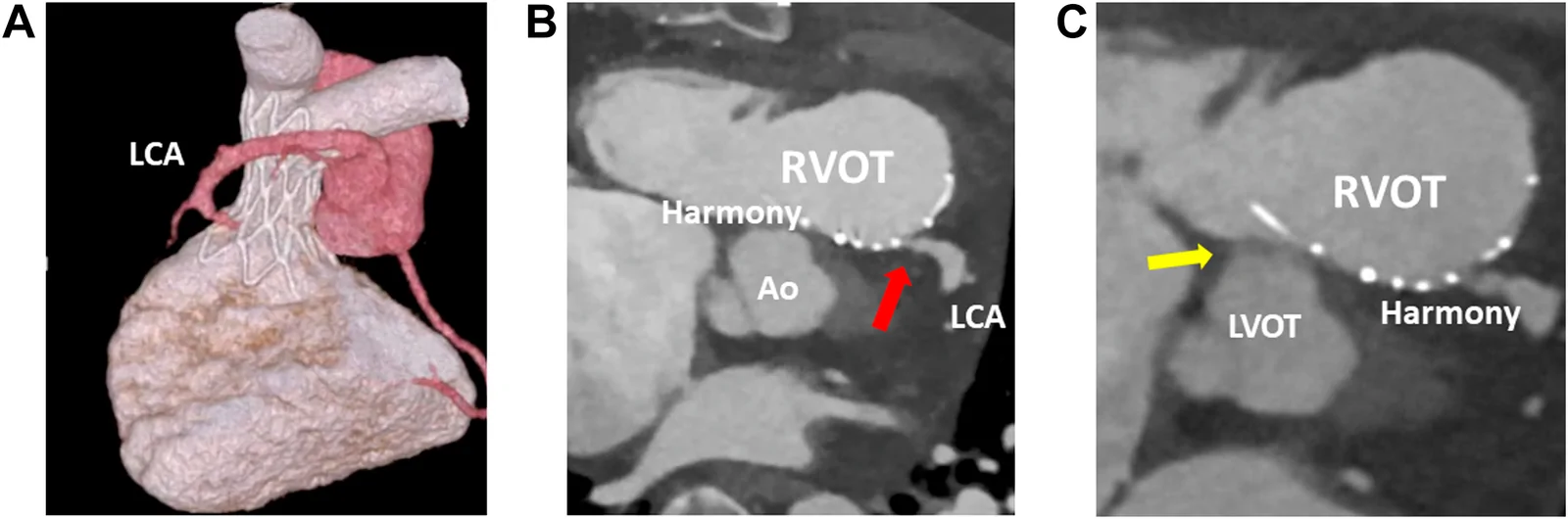
Postprocedural Cardiac Computed Tomography Findings (A and B) A coronary artery–right ventricular fistula adjacent to the implanted valve (red arrow). (C) Residual VSD located near the prosthetic valve structure (yellow arrow). Ao = aorta; LCA = left coronary artery; LVOT = left ventricular outflow tract; RVOT = right ventricular outflow tract.

**Figure 6:**
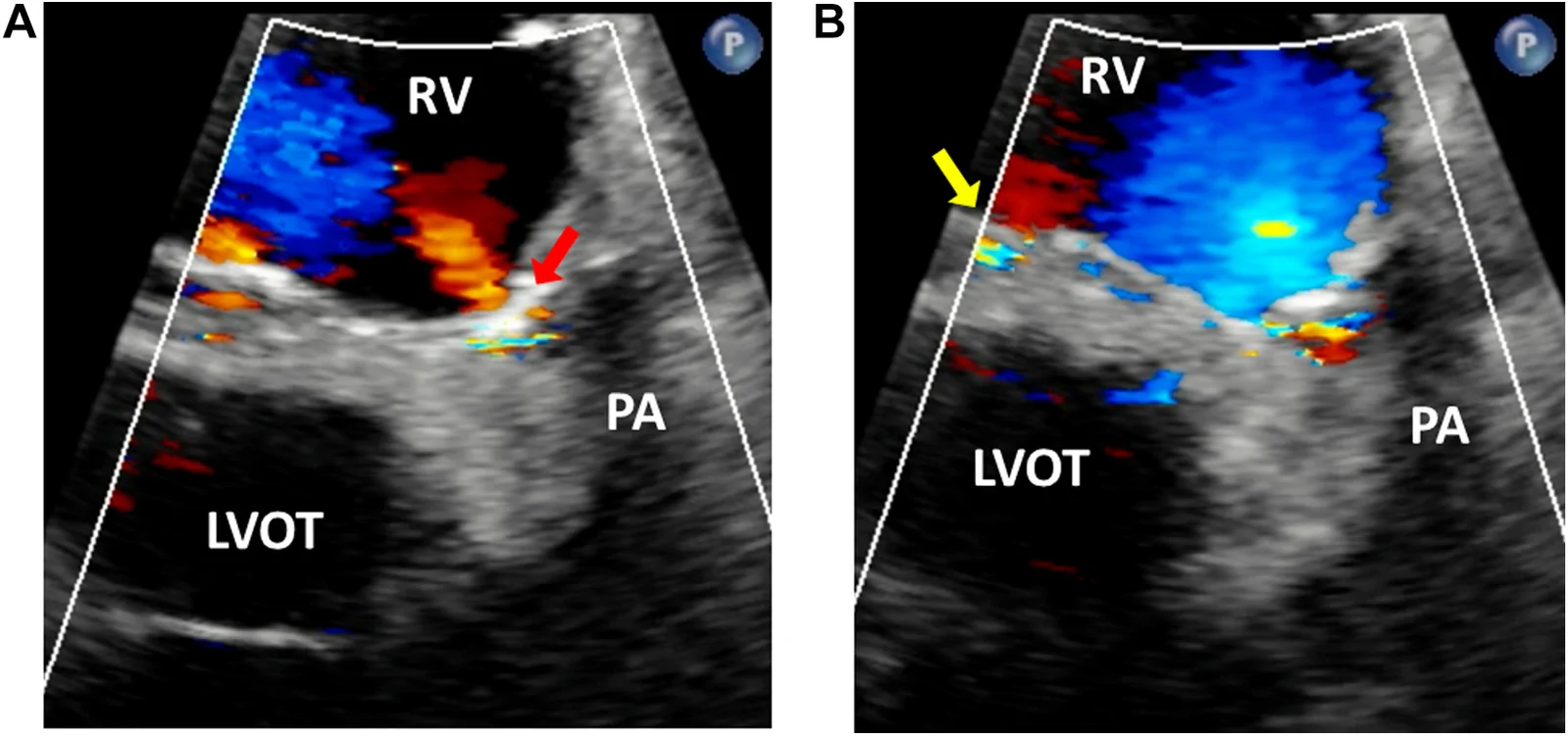
Postprocedural Transchoracic Echocardiography Findings (A) A coronary artery–right ventricular fistula adjacent to the implanted valve (red arrow). (B) Residual ventricular septal defect (VSD) located near the prosthetic valve structure (yellow arrow). LVOT = left ventricular outflow tract; PA = pulmonary artery; RV = right ventricle.

Hemolytic findings relatively improved with blood pressure adjustment, and the patient was discharged on postoperative day 8. Because endothelialization of the implanted valve and spontaneous reduction of shunt flow were anticipated, invasive intervention was deferred. After discharge, the patient remained free from adverse event-related hospitalization and was followed in the outpatient clinic at 10 days, 1 month, and 3 months. Serial laboratory testing demonstrated no increase in bilirubin levels or lactate dehydrogenase or progression of anemia. If laboratory evidence of persistent hemolysis fails to improve during follow-up, transcatheter closure of the coronary artery–right ventricular fistula or surgical closure of the residual ventricular septal defect will be considered.

## Discussion

### TPVI in repaired TOF

Patients with repaired TOF, particularly those who underwent transannular patch repair, frequently develop chronic pulmonary regurgitation during long-term follow-up.^3^ Progressive pulmonary regurgitation may result in right ventricular dilation, ventricular arrhythmias, exercise intolerance, heart failure, and sudden cardiac death.^5^ TPVI has emerged as a less invasive alternative to surgical pulmonary valve replacement and has increasingly been applied, particularly in patients with large or patch-repaired RVOTs, who often have a history of multiple surgeries, using self-expandable valves such as the Harmony valve.^6^

### Hemolysis after TPVI

Mechanical hemolysis is a recognized complication of transcatheter valve interventions. Excessive shear stress, turbulent flow, prosthetic surface interaction, and paravalvular leakage are considered major mechanisms of red blood cell destruction. However, most reported cases involve left-sided valvular interventions because the systemic circulation generates higher pressure gradients and flow velocities.^9^

Conversely, clinically significant hemolysis after TPVI is extremely rare. The right-sided circulation is generally a low-pressure system, and trivial transvalvular or paravalvular leakage alone is unlikely to produce sufficient shear stress to induce severe hemolysis.^9^^,^^10^ Therefore, additional hemodynamic factors were considered important in the present case.

### Potential role of the left-right shunt

In the present patient, postoperative imaging demonstrated both a coronary artery–right ventricular fistula and a residual VSD adjacent to the implanted valve. Although the relative contribution of each lesion cannot be determined, we speculate that both generated high-velocity shunt flow toward the prosthetic valve, resulting in localized turbulence and excessive shear stress sufficient to cause mechanical hemolysis.

Although the residual VSD was present before TPVI, no clinically significant hemolysis had been observed. We speculate that implantation of the Harmony valve fundamentally altered the local flow pattern by introducing a prosthetic frame into the RVOT. The high-velocity shunt jets from the residual VSD and coronary artery–right ventricular fistula likely impinged on the valve frame, generating localized turbulence and excessive shear stress sufficient to cause mechanical hemolysis.^9^ Incomplete endothelialization immediately after valve implantation may have enhanced the interaction between circulating blood cells and the prosthetic surface.^11^ Additionally, implantation of the prosthetic valve may have altered the geometry and direction of the preexisting shunt jets, creating a new interaction between the high-velocity flow and the valve frame that was not present before TPVI. Although speculative, this altered flow pattern may further explain why clinically significant hemolysis developed only after valve implantation.

### Importance of preprocedural imaging

Current preprocedural assessment before TPVI mainly focuses on RVOT anatomy, pulmonary valve morphology, coronary compression risk, and landing zone suitability. However, the present case suggests that, in addition to the conventional assessment, careful evaluation of residual shunt lesions around the RVOT may also be clinically important. In the present case, although residual VSD had been recognized before TPVI, its potential interaction with the implanted valve as a source of localized high-shear stress was not fully appreciated before the procedure, and the coronary artery–right ventricular fistula was identified only after TPVI during the evaluation of hemolysis. Coronary artery compression is a well-recognized complication of TPVI, particularly in patients with anomalous coronary artery anatomy. No evidence of coronary compression was observed in the present case.

Careful multimodality imaging assessment may help identify residual VSDs, coronary fistulas, or other abnormal shunt pathways that could interact with the implanted valve. Supraannular positioning may, for instance, prevent interference between the implanted valve and VSD. Prophylactic blood pressure management may be attempted following TPVI in such high-risk patients. Device occlusion of these shunts may be attempted for refractory hemolysis, if applicable.

## Conclusions

We experienced a rare case of mechanical hemolysis after successful TPVI in a patient with repaired TOF. Residual left-to-right shunts adjacent to the RVOT were considered possible contributors to localized turbulent flow and mechanical hemolysis after valve implantation. Careful multimodality assessment of residual shunt lesions before TPVI may help identify patients at risk for this rare complication.

**Take-Home Messages**

• Residual left-to-right shunts adjacent to the right ventricular outflow tract may contribute to mechanical hemolysis after Transcatheter pulmonary valve implantation.
• Multimodality imaging before Transcatheter pulmonary valve implantation may help identify high-risk residual lesions in patients with repaired congenital heart disease.

**Visual Summary:** Timeline of the Case

Period	Clinical events
Childhood	TOF repair with transannular patch
Adulthood	Progressive pulmonary regurgitation and RV enlargement
Hospital admission	Planned TPVI for symptomatic PR
Day 0	Successful Harmony TPVI implantation
Postoperative day 1	Dark-red urine and laboratory hemolysis
Postoperative day 2-3	CT and Echo identified residual VSD and coronary artery–RV fistula
Conservative management	Antihypertensive therapy for shear stress reduction
Postoperative day 8	Hemolysis improved and discharged

CT = computed tomography; PR = pulmonary regurgitation; RV = right ventricle; TOF = Tetralogy of Fallot; TPVI = transcatheter pulmonary valve implantation; VSD = ventricular septal defect.

## Funding Support and Author Disclosures

The authors have reported that they have no relationships relevant to the contents of this paper to disclose.
